# Understanding how discrimination can affect health

**DOI:** 10.1111/1475-6773.13222

**Published:** 2019-10-29

**Authors:** David R. Williams, Jourdyn A. Lawrence, Brigette A. Davis, Cecilia Vu

**Affiliations:** ^1^ Department of Social and Behavioral Sciences Harvard T.H. Chan School of Public Health Boston Massachusetts; ^2^ Department of African and African American Studies Department of Sociology Harvard University Cambridge Massachusetts

**Keywords:** discrimination, health, health disparities, mental health, racism

## Abstract

**Background:**

To provide an overview of the empirical research linking self‐reports of racial discrimination to health status and health service utilization.

**Methods:**

A review of literature reviews and meta‐analyses published from January 2013 to 2019 was conducted using PubMed, PsycINFO, Sociological Abstracts, and Web of Science. Articles were considered for inclusion using the Preferred Reporting Items for Systematic Review and Meta‐Analyses (PRISMA) framework.

**Results:**

Twenty‐nine studies met the criteria for review. Both domestic and international studies find that experiences of discrimination reported by adults are adversely related to mental health and indicators of physical health, including preclinical indicators of disease, health behaviors, utilization of care, and adherence to medical regimens. Emerging evidence also suggests that discrimination can affect the health of children and adolescents and that at least some of its adverse effects may be ameliorated by the presence of psychosocial resources.

**Conclusions:**

Increasing evidence indicates that racial discrimination is an emerging risk factor for disease and a contributor to racial disparities in health. Attention is needed to strengthen research gaps and to advance our understanding of the optimal interventions that can reduce the negative effects of discrimination.

## 1 INTRODUCTION

Racial and ethnic differences in health, in which socially disadvantaged racial populations have worse health than whites, are large, pervasive across a broad range of outcomes, and persistent over time.^1^ They exist for the onset of disease, as well as the severity and course of illness. Socioeconomic status (SES)—whether measured by income, education, occupational status, or wealth—is a strong predictor of variations in health and has often been viewed as the driver of racial inequities in health. Research finds that although SES predicts variations in health status within each racial group, racial disparities persist at every level of SES.^2^ There is a large and growing body of empirical evidence indicating self‐reports of discrimination are race‐related aspects of social experience that can have negative effects on health. This paper provides an overview of research on self‐reported discrimination and health, as well as health care utilization. It begins by situating research on racial discrimination and health within the larger context of research on racism and health. Importantly, self‐reported experiences of discrimination are one mechanism by which racism affects health, and these exposures can be best understood and effectively addressed within the context of the role of racism in health. The paper then highlights key findings in this burgeoning literature.

## 2 BACKGROUND AND THEORETICAL FRAMEWORK

**Figure 1:**
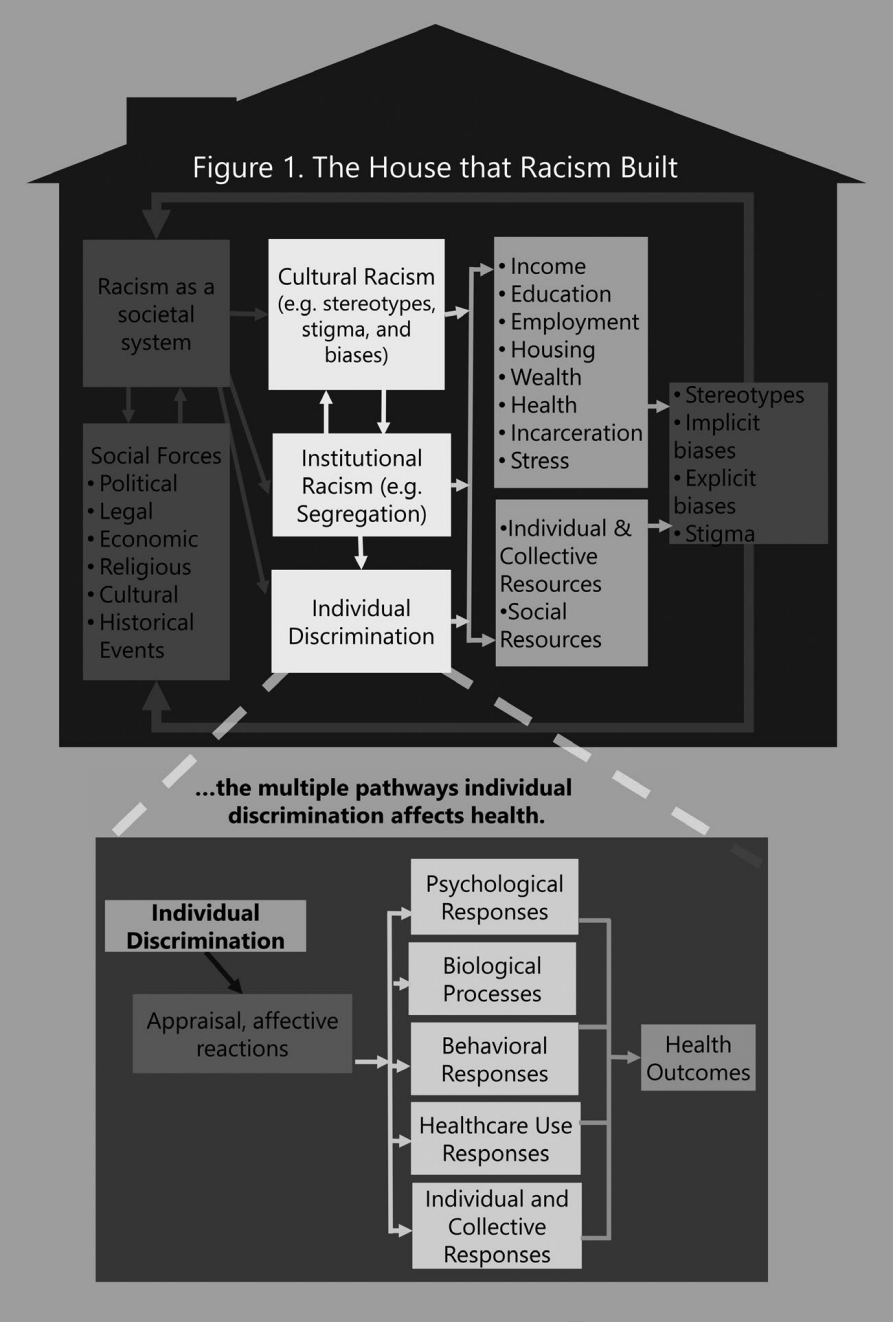
The House that Racism Built

Figure 1 illustrates the multiple components of racism and the ways in which these components can affect health. Racism is viewed as a dynamic societal system that is shaped by and reshapes other social institutions such as the political, legal, and economic systems.^3^, ^4^, ^5^, ^6^ Central to racism, in the US context, is a hierarchical ideology that the dominant white group uses to categorize and rank social groups into races with whites being superior compared to other races. There are three major pathways that link racism to inequities in society and health. The first pathway by which racism operates is cultural racism.^6^ This refers to the embedding of the inferiority of blacks and other nonwhites into the belief systems, images, and norms of the larger culture that leads to widespread negative beliefs (stereotypes) and attitudes (prejudice) that devalue, marginalize, and subordinate nonwhite racial populations. Cultural racism creates a larger ideological environment within which the system of racism can flourish. It initiates and sustains racial prejudice and negative racial stereotypes that can lessen support for egalitarian policies, trigger health‐damaging psychological responses in stigmatized persons such as internalized racism and stereotype threat, and facilitate explicit and implicit biases that restrict access to desirable resources, including medical care.^6^

The second pathway is institutional or structural racism. We use these terms interchangeably to refer to societal structures and policies that reduce access of the socially stigmatized to desirable opportunities and resources in society.^5^ The system of racism develops and sustains policies and structures that empower the dominant group to differentially allocate desirable societal opportunities and resources to racial groups regarded as inferior. Residential segregation is one example of an institutional mechanism of racism that adversely affects health in multiple ways.^7^, ^8^ The forced removal and relocation of American Indians to reservations is another example of institutionalized isolation of a marginalized racial population. Segregation is a critical determinant of SES, as it reduces access to quality elementary and high school education, preparation for higher education, and access to employment opportunities. One national study found that the elimination of segregation would erase black‐white differences in income, education, and unemployment, and reduce racial differences in single motherhood by two‐thirds.^9^ SES, in turn, is a strong predictor of variation in health and risk factors that affect health. Segregation can also lead to increased exposure to multiple psychosocial, physical, and chemical stressors linked to neighborhood and housing conditions, including crime, violence, and air pollution. It can also affect access to and the quality of local services, ranging from medical care to municipal services.

The third pathway through which racism operates is through individual‐level discrimination. Stigmatized racial groups experience differential treatment (discrimination) directed at them by both social institutions and individuals. Considerable scientific evidence documents the persistence of objectively assessed individual discrimination in contemporary society. A review of audit studies—those in which researchers carefully select, match, and train individuals to be equally qualified in every respect but to differ only in race—provide striking examples of contemporary racial discrimination.^10^ Discrimination has been documented in renting apartments, purchasing homes and cars, obtaining mortgages and medical care, applying for insurance, and hailing taxis. Such incidents of discrimination can lead to reduced access to a broad range of societal resources and opportunities. Figure 1 indicates that the persistence of stark racial inequities in multiple domains of society can confirm racial stereotypes and stigma, and thus serve to reinforce the system of racism. Moreover, the pathways by which racism affect are interrelated and mutually reinforcing.^11^

The lower panel of Figure 1 serves to further unpack how individual‐level discrimination can affect health. The focus here is on a subset of incidents of individual discrimination that is perceived by the individual. According to social stress theory, perceived discrimination is a type of stressor that, like other psychosocial stressors, is adversely related to a broad range of physical and mental health outcomes.^12^, ^13^ A recent study, for example, documented that self‐reported experiences of discrimination are associated with neural functioning in ways that mirror patterns observed for other psychosocial stressors (eg, greater spontaneous amygdala activity and greater connectivity between the amygdala and other regions of the brain including the thalamus).^14^ The lower panel of Figure 1 delineates how discriminatory incidents of which the individual is aware can trigger appraisal and affective reactions that can be experienced as stressful life exposures, and they have a cascade of negative effects on health.^15^ They can lead to negative emotions that can adversely affect psychological well‐being, leading to symptoms of distress and increasing the risk of discrete psychiatric disorders. These negative emotions can also lead to biological dysregulation that can contribute to indicators of subclinical disease and chronic physical illness.^15^ Coping with negative emotional states can also lead to increases in risky health behaviors, including declines in the utilization of and engagement with health care services. Figure 1 also acknowledges that in the face of exposure to discrimination, individuals and groups can respond in ways that can neutralize at least some of the negative effects of discrimination.

## 3 METHODS

### 3.1 Search strategy

Reviews were identified through a search of PubMed, PsycINFO, Sociological Abstracts, and Web of Science. Reviews were eligible for inclusion if they were focused reviews or meta‐analyses, in English, published from January 2013 to the present, extending the systematic review and meta‐analysis published by Paradies and colleagues.^16^ The following keywords were used: (racism* OR social discrimination*) OR (race* OR racial*) AND discriminat*)) AND (systematic*[sb] OR systematic*[ti] OR review*[ti] OR review*[sb] OR meta‐analysis*[ti]). The bibliographies of included studies were manually examined to identify additional reviews and meta‐analyses.

### 3.2 Inclusion criteria

Two of us (JAL, CV) reviewed titles and abstracts of the traced articles followed by a full‐text review to check inclusion criteria using the Covidence systematic review software.^17^ A third author (DRW) acted as a tiebreaker regarding study selection and inclusion. A review was eligible for inclusion if it satisfies the following criteria: (a) evaluated studies examining self‐reported racial/ethnic discrimination or studies that examined perceived discrimination broadly, and (b) examined health or health‐related outcomes. This is consistent with the finding that adverse health effects of discrimination are generally evident, irrespective of whether an incident is linked to a general perception of bias or unfair treatment or to discriminatory experiences attributed to race/ethnicity or other stigmatized social statuses.^18^, ^19^ The outcomes were mental health, including positive psychological well‐being, indicators of physical health and risk factors, health behaviors, and health service utilization.

## 4 RESULTS

Of 1189 articles screened, based on the criteria for inclusion, two authors (JAL, CV) completed title and abstract screening for 922 unique studies, identifying 32 for full‐text review. An additional study was identified for inclusion (n = 33) from a review of bibliographies. A total of 29 reviews were extracted for analysis (Table 1).

**Table 1:** Reviews of the research literature linking discrimination and health

Discrimination study	Focus	No. papers included	Study design	Health outcomes	Findings
Mental health					
Britt‐Spells, AM., et al (2018)	Depressive symptoms	12	Cross‐sectional: 100%	Depressive symptoms, psychological distress, psychiatric symptoms	Positive (*r* = .290; 95% CI: 0.235, 0.343)
Carter, RT., et al (2019)	Mental and physical health	242 Mental: 200 Physical: 48 Cultural: 88 Substance use: 23	Cross‐sectional Longitudinal*	Adverse mental health (eg, anxiety, depression, hostility, anger, stress, psychological distress), physical health (eg, blood pressure, BMI, self‐reported health), and substance use (eg, alcohol, smoking, polysubstance use) outcomes	Overall: positive (*r* = .16, *P* < .01) Mental: positive (*r* = .21, *P* < .01) Substance use: positive (*r* = .16, *P* < .01) Physical: positive (*r* = .07, *P* < .01)
Carter, RT., et al (2017)	Mental and physical health	105	Cross‐sectional Longitudinal*	Adverse mental health, physical health, and substance use	Overall: positive (*r* = .17; 95% CI: 0.15, 0.20) Mental: positive (*r* = .20; 95% CI: 0.17, 0.24) Physical: positive (*r* = .09; 95% CI: 0.03, 0.14) Substance use: null (*r* = .12; 95% CI: −0.02, 0.25)
de Freitas, DF., et al (2018)	Mental health	51	Cross‐sectional Longitudinal*	Psychological disturbance, depression, anxiety, psychosis, perceived stress, externalizing behavior, self‐esteem, positive evaluation of life, self‐efficacy, well‐being psychological adaptation	Overall: positive (*r* = .17; 95% CI: 0.15, 0.20) Mental: positive (*r* = .20; 95% CI: 0.17, 0.24) Physical: positive (*r* = .09; 95% CI: 0.03, 0.14) Substance use: null (*r* = .12; 95% CI: −0.02, 0.25)
Hopkins, PD., Shook, NJ. (2017)	Anxiety	24	Cross‐sectional	General anxiety disorder or social anxiety disorder	Positive: 100% (n = 3) One study found a positive association with general anxiety disorder among a sample of only African Americans
Jones, KP., et al (2016)	Mental and physical health	90	Of the 44 primary samples obtained, 4/44 were from experimental studies	Psychological health, physical health	Positive: physical health (*r* = .19; 95% CI: 0.07‐0.21); psychological health (*r* = .30; 95% CI: 0.15‐0.36)
Kirkinis, K., et al (2018)	Trauma	28 papers 44 associations	Cross‐sectional: 93% (n = 26) Longitudinal: 7% (n = 2)	PTSD, dissociation, other measures of trauma, and race‐based traumatic stress symptoms	Positive: 70% (31/44 associations)
Lewis et al (2015)	Mental disorders	12	Cross‐sectional	DSM‐IV disorders (depression, anxiety, eating, psychotic)	Positive association with disorders Inverse association with depression, anxiety for Asian immigrants
Paradies, Y., et al (2015)	Mental and physical health	293	Cross‐sectional: 89.8% Longitudinal: 9.0% Other: 1.2%	Negative mental health (ie, depression, distress, stress, anxiety, internalizing, negative affect, PTSD, somatization, suicide ideation/ attempts, other mental health symptoms, general mental health, overall negative mental health); positive mental health (ie, self‐esteem, control, life satisfaction, positive affect, well‐being, overall positive mental health); physical health (ie, BP, heart conditions, overweight, diabetes, misc.); general health	Negative: negative mental health (*r* = −.23; 95% CI: −0.24, −0.21); positive mental health (*r* = −.13; 95% CI: −0.16, −0.10); general health (*r* = −.13; 95% CI: −0.18, −0.09); physical health (*r* = −.09; 95% CI: −0.12, −0.06)
Potter, LN., et al (2019)	Daily mental health	25	Longitudinal (daily diary studies)	Poor mental health in daily life (eg, depressive symptoms, negative affect, somatic symptoms, active coping)	Positive: poor mental health (10/11; 91%)
Schmitt, MT., et al (2014)	Psychological well‐being	328 for the first research question and 54 for the second	Cross‐sectional and longitudinal for the first meta‐analysis and experimental studies only for the second meta‐analysis	Psychological well being, broadly constructed (ie, mood, self‐esteem, anxiety, depression, life satisfaction, affect, other measures of mental health)	Negative: psychological well‐being (*r* = −.21; 95% CI: −0.22, −0.19)
Triana, MC., et al (2015)	Mental and physical health	79	Cross‐sectional Longitudinal*	Psychological health (ie, stress, mental health, anxiety, negative affect, self‐esteem, life satisfaction, and depression); physical health (ie, blood pressure, bodily pain, general physical health, illness, drug or alcohol use)	Negative: psychological health (*r* = −.12, *ρ* = −0.14); physical health (*r* = −.06, *ρ* = −0.07) Positive: coping behavior (*r* = .17, *ρ* = 0.20)
Vines, AI., et al (2017)	Mental health	85	Cross‐sectional Longitudinal*	Mental health was not specified, but includes PTSD, depression. mediators/ confounders: aggression, coping & personality, internalized psych response (eg, self‐esteem), external supportive buffers	Conditional/mixed: no percentage breakdown of trends
Physical health					
Bernardo, CD., et al (2017)	Adiposity	10	Longitudinal: 100%	Weight change; waist circumference change; BMI change; become obese; remain obese	Weight change: positive Waist circumference change: 1 positive, 3 null BMI change: 2 positive, 2 null Become obese: positive Remain obese: null
Black, LL., et al (2015)	Physical health	19	Cross‐sectional Longitudinal*	Heart disease risk factors (ie, CRP (C‐reactive protein); coronary calcium positive status; IMT (carotid intima‐media thickness); arterial plaque; coronary artery calcification); blood pressure (and incidence of hypertension); adverse birth outcomes; cancer/tumor incidence; weight change (and body fat distribution); other outcomes (ie, all‐cause mortality (ACM); Epstein‐Barr virus reactivation (EBV); frequency of common colds/physical illnesses (cold))	Heart disease risk factors: null (3/3) Blood pressure: null (3/3) Adverse birth outcomes: null (1/6); positive (5/6) Cancer/tumor incidence: conditional on context of discrimination (1/2); positive (1/2) Weight change: positive (1/2); negative (1/2) Other health outcomes: ACM: null (1/1); EBV: positive (1/1); Cold: positive (1/1)
Busse, D., et al (2017)	Stress	27	Experimental: 37% (n = 10) Longitudinal: 7% (n = 2) Cross‐sectional: 56% (n = 15)	Hypothalamic‐pituitary‐adrenal (HPA) axis: salivary and awakening cortisol; dehydroepiandrosterone (DHEA); corticotropin‐releasing hormone	Salivary cortisol: 1/2 positive; 1/2 negative Cortisol awakening response: 1/1 positive Null: corticotropin‐releasing hormone; afternoon DHEA
Dolezsar, CM., et al (2014)	Hypertension	44	Cross‐sectional, longitudinal, experimental designs*	Hypertensive status; blood pressure	Positive: hypertensive status (*z* = 0.05; 95% CI: 0.01, 0.09), nighttime ambulatory blood pressure (*z* = 0.15; 95% CI: 0.04, 0.19) Null: blood pressure (systolic: *z* = 0.01; 95% CI: −0.01, 0.03) (diastolic: *z* = 0.2; 95% CI: −0.01, 0.03)
Korous, KM., et al (2017)	Cortisol	16	Experimental: 25% (n = 4) Nonexperimental: 75% (n = 12)	Current cortisol, diurnal cortisol, cortisol reactivity, average cortisol	Positive (*r* = .040; 95% CI: 0.038‐0.117)
Lewis, TT., et al (2014)	Cardiovascular health	38	26 cross‐sectional 12 longitudinal/cohort or unspecified	Lifestyle factors (eg, smoking, physical activity, alcohol intake); hypertension and blood pressure; biomeasures (eg, obesity, C‐reactive protein, coronary artery occlusion)	Conditional: lifestyle factors; resting blood pressure/hypertension; biomeasures Positive: ambulatory blood pressure
Lockwood, KG., et al (2018)	Cardiovascular health	21	Cross‐sectional, longitudinal, experimental designs*	Cardiovascular reactivity (ie, blood pressure, heart rate, heart rate variability, total peripheral resistance, preejection period, cardiac output); HPA axis (ie, diurnal cortisol slope); immune (ie, C‐reactive protein, interleukin, monocyte chemoattractant protein, tumor necrosis factor, interferon), neural activity	Generally positive associations for CVD reactivity, flatter diurnal cortisol slopes, systemic inflammation, and neural activity in the brain regions consistent with exposure to psychosocial stress
Health behaviors					
Desalu, JM., et al (2019)	Alcohol use	27	Cross‐sectional: 85% Longitudinal: 15%	Consumption; binge/heavy drinking; at‐risk drinking; alcohol use disorders (AUD); negative drinking consequences	Positive: consumption (*r* = .12; 95% CI: 0.08, 0.17); binge drinking (*r* = .06; 95% CI: 0.02, 0.10); at‐risk drinking (*r* = .14; 95% CI: 0.06, 0.23); negative drinking consequences (*r* = .25; 95% CI: 0.09, 0.42) Null: AUD (*r* = .10; 95% CI: −0.01, 0.20)
Gilbert, PA., et al (2016)	Alcohol use	97	Cross‐sectional: 80% Longitudinal: 18% Experimental: 2%	Alcohol‐related outcomes (number of drinks per month, past 2 weeks of binge drinking, past week/ 30 days/ year of alcohol use, past 2 months of weekend drinking, drinking‐related problems, past‐year alcohol use, past 30/90 days of binge drinking, drinking debut, alcohol use disorder, lifetime alcohol use, hazardous drinking, current alcohol use, alcohol use disorder)	Positive: 45% (n = 14) Null: 32% (n = 10) Conditional: 23% (n = 7)
Slopen, N., et al (2016)	Sleep	17	Longitudinal: 29% (n = 5 [1 daily diary]) Cross‐sectional: 71% (n = 12)	Poor sleep outcomes (ie, duration, efficiency, sleep latency, wake after sleep onset, REM sleep, light sleep, stage 3 and 4 sleep)	Positive: sleep difficulties or insomnia (16/16; 100%); poor sleep quality (7/7; 100%)
Health care utilization					
Ben, J., et al (2017)	Health care utilization	Review: 83 Meta‐analysis: 59	Cross‐sectional: 96.4% Longitudinal: 3.6%	Health service experiences [HSE] (ie, communication; satisfaction/perceived quality of care; trust; some combination of these) Health service utilization [HSU] (ie, having examinations, screenings, checks, etc; uptake of treatments, medications, vaccinations; hospital visits and admissions to ERs; delaying health care; insurance coverage; some combination of these)	HSE: negative HSU: conditional on outcome, negatively associated with uptake of treatments and seeking health care; no association for the other measures
Gaston, GB., et al (2013)	HIV treatment adherence	16	Qualitative	Antiretroviral medication or medical self‐care adherence	Discrimination serves as a barrier to medical care, poorer self‐rated health, lower self‐care adherence, less satisfaction with care, greater depressive symptoms
Children and adolescents					
Alhusen, JL., et al (2016)	Maternal and child health	15	Qualitative: 27% (n = 4) Quantitative: 73% (n = 11)	Preterm birth; low birth weight; small‐for‐gestational‐age newborn; access to and quality of prenatal care	Preterm birth (quant studies: 5): 3/5 null; 2/5 positive Low birth weight (quant studies: 3): 2/3 positive; 1/3 null Small‐for‐gestational‐age: positive Initiation of prenatal care (quant studies:1): null
Benner AD. et al (2018)	Socioemotional, academic, and behavioral health	214	Cross‐sectional Longitudinal*	Socioemotional well‐being (depression, internalizing symptoms, positive well‐being, self‐esteem); academic (achievement, school engagement, motivation); behavioral (externalizing behaviors, risky sex behaviors, substance use, deviant peer affiliations)	Racial discrimination was positively associated with depression, internalizing symptoms, externalizing behaviors, risky sex behaviors, substance use, deviant peer affiliations and negatively associated with self‐esteem, academic achievement, school engagement, academic motivation
Heard Garris NJ et al (2018)	Child health (infant health outcomes, mental health, socioemotional health, health care utilization, physical health, cognitive development, and youth health)	30	Case‐control (10%), cross‐sectional (27%), and longitudinal (53%)	Infant health outcomes (preterm birth, cortisol reactivity, birthweight); mental health (depressive symptoms, anxiety, substance use, well‐being, anxiety); socioemotional health (externalizing and internalizing behavior, socioemotional difficulties, self‐esteem, positive behavior); health care utilization (frequency of sick child visits); physical health (BMI, general child illness, weight for age); cognitive development (spatial ability); youth health outcomes (depressive)	Caregiver racial discrimination is associated with preterm birth in 4/7 studies, cortisol reactivity in 1/1 study, and birthweight in 6/9 studies observing child outcomes. Within postbirth, caregiver pathway: caregiver racial discrimination is associated with depressive symptoms in 1/7 studies, anxiety in 1/3, substance use in 1/2, well‐being in 1/1, depressive symptoms in 1/7, externalizing in 7/10, internalizing behavior in 4/7, socioemotional difficulties in 2/2, self‐esteem in 1/1, positive behavior in 1/4, frequency of sick child visits in 1/2, BMI in 1/1, general child illness in 1/2, weight for age in 1/1, and spatial cognitive ability in 1/1 in child outcomes; within postbirth, other pathway: caregiver racial discrimination was associated with depressive symptoms in 1/2 studies in child outcomes
Priest N. et al (2013)	Mental and physical health (negative and positive mental health, negative and positive general health, physical health, negative and positive pregnancy, behavior problems, well‐being, health‐related behaviors, health care utilization)	121	2% Case‐control, 78% Cross‐sectional, and 20% longitudinal	Negative mental health (anxiety, depression, distress, hopelessness, loneliness, negative self‐esteem, posttraumatic stress, psychological distress, social and emotional difficulties, somatic symptoms, stress, suicide, mental health problems); positive mental health (emotional adjustment, psychological adjustment, psychological adaption, resilience, self‐esteem, self‐worth, social and adaptive functioning); physical health (blood pressure, childhood illnesses, common childhood illnesses, insulin resistance, obesity, physical symptoms); general health; negative general health (feeling unhappy, feeling unhealthy, health problems); positive general health (self‐rated health); well‐being (general health, HrQoL, life satisfaction, well‐being); negative pregnancy (LBW, preterm birth, VLBW); positive pregnancy (birth weight, gestational age); behavior problems (ADHD, aggression, behavior problems, conduct problems, delinquent behavior, deviance, emotional and behavioral problems, externalizing, internalizing, problem behavior; health‐related behavior (alcohol, drug use, smoking); health care utilization (access and cost)	Of the 121 studies and 461 associations, 46% of associations were negatively associated with reported racial discrimination, 18% were positive and 3% were conditional. 76% of the associations between racial discrimination and negative mental health outcomes were positive. 62% of the associations between racial discrimination and negative mental health outcomes were negative. 69% of the associations between racial discrimination and behavior problems/delinquent behaviors were positive. 51% of the associations between racial discrimination and health‐related behaviors were positive. 45% of the associations between racial discrimination and well‐being/life satisfaction/quality of life outcomes were negative and 50% was unrelated. 79% of the associations between racial discrimination and negative pregnancy/birth outcomes were positive. 67% of the associations between racial discrimination and physical health had no significant associations. Additionally, mental health was the most studied association among the 121 studies and 461 relationships (51% of associations were mental health‐related)

* Study type breakdown was not specified.

### 4.1 Discrimination and mental health

A 2015 meta‐analysis by Paradies and colleagues^16^ found over 300 articles on racial discrimination and health published through 2013, with the association between discrimination and mental health stronger than for physical health. Although 8 out of every 10 studies came from the United States, there were publications from 19 other countries. Discrimination was significantly associated with poorer mental health outcomes (eg, depression, anxiety, psychological stress, *r* = −.23) and positive mental health outcomes (eg, self‐esteem, life satisfaction, control, well‐being, *r* = −.13). The meta‐analysis found that the effect sizes for the association between perceived discrimination and mental health were stronger in cross‐sectional studies than in longitudinal ones and in nonrepresentative samples than in representative ones.

A meta‐analysis of 51 studies in Europe highlights growing international evidence. Across diverse ethnic populations, positive associations were found between ethnic discrimination and emotional distress, as well as inverse associations with positive markers of well‐being, such as self‐esteem and self‐efficacy.^20^ Several recent reviews continue to document an inverse association between discrimination and good mental health.^21^, ^22^, ^23^, ^24^, ^25^, ^26^, ^27^ For example, a 2014 review reported the results of two meta‐analyses focused on the association between discrimination and well‐being.^28^ Discrimination, in the first meta‐analysis, was associated with poorer well‐being (self‐esteem, depressive and anxiety symptoms, psychological distress, and life satisfaction), with the association being somewhat weaker for positive outcomes than negative ones. The observed associations (effect sizes) were larger for disadvantaged groups compared to advantaged groups (eg, women vs men) and for children than for adults. They were also evident in both cross‐sectional and longitudinal analyses. In the second meta‐analysis, the researchers examined experimental data for studies relating the manipulation of discrimination to indicators of well‐being. The study found a significant negative effect (*d* = −0.25) of multiple exposures to discrimination on well‐being. A single event of discrimination was not adversely related to well‐being. Research also indicates that exposure to discrimination can adversely affect the personality characteristics of adults. Longitudinal analyses in two national studies, the Health and Retirement Survey and the Midlife in the United States Study (MIDUS), found that incident discrimination was associated with increases in neuroticism (negative emotions) and declines in agreeableness (trusting) and in conscientiousness (organization and discipline).^29^

One review documented that in addition to discrimination being positively associated with measures of depression, anxiety symptoms, and psychological distress, it is also associated with increased risk of defined psychiatric disorders.^18^ For example, in the National Study of American Life (NSAL), among African American and Caribbean Black adults 55 years and older, both racial and nonracial chronic Everyday Discrimination was positively associated with increased risk of any lifetime (LT) disorder, as well as LT mood and anxiety disorders.^30^ It was also associated with an increased risk of depressive symptoms and serious psychological distress. Similarly, in the National Latino and Asian American Study (NLAAS), Everyday Discrimination was associated with an increased risk of psychiatric disorders, but the association was stronger among Mexicans than for Puerto Ricans.^31^ In the same study, Everyday Discrimination was associated, in multivariate models, with increased odds of any DSM‐IV disorder (odds ratio [OR] = 1.90), depressive disorder (OR = 1.72), and anxiety disorder (OR = 2.24) among Asian Americans.^32^ Another review documented a positive association between discrimination and PTSD or other indicators of trauma in 70 percent of the associations examined.^33^

Research also reveals that the accumulation of experiences of discrimination over time is associated with an increased risk of mental health problems. For example, in the Study of Women Across the Nation (SWAN), the levels of Everyday Discrimination were assessed six times over 10 years.^34^ It found that women who experienced the highest accumulation of experiences of discrimination over time, domains, and attributes (race/ethnicity, sex, or other) reported the highest levels of depressive symptoms. This pattern was evident for all women (black, Chinese, Hispanic, and white), regardless of their race or ethnic group. Similarly, a study in the United Kingdom examined the cumulative, longitudinal effects of racial discrimination on mental health of ethnic minorities.^35^ The study found evidence of a dose‐response relationship between the cumulative discrimination measure (number of experiences and number of time points exposed) and a scale of nonspecific psychological distress.

Most of the early studies of discrimination were cross‐sectional. In addition, the extent to which observed associations between discrimination and mental health outcomes were due to unmeasured psychological factors remained unclear. These concerns have been addressed in recent research.^18^ Although the majority of studies of discrimination and health are still cross‐sectional, there are a growing number of prospective studies that link changes over time in discrimination to increases in symptoms of distress and depression. One review of 25 daily diary, longitudinal studies found that over 90 percent of the time, discriminatory events on a given day were associated with increased symptoms of distress.^36^ A few studies have also documented that the association between discrimination and mental health remains robust after adjustment for potential psychological confounders such as neuroticism, social desirability, hostility, and negative affect.^18^

### 4.2 Discrimination and physical health

In the Paradies meta‐analysis model, racial discrimination was significantly associated with poorer general health (*r* = −.13) and poorer physical health (*r* = −.09).^16^ Research also reveals that discrimination is associated with multiple indicators of adverse cardiovascular disease (CVD) outcomes and risk factors of CVD. A 2014 paper^37^ reviewed the research on self‐reported discrimination and CVD published between 2011 and 2013. It found that most studies focused on hypertension, smoking, and other health behaviors, with few studies on cardiovascular endpoints. However, one study documented that self‐reported discrimination was associated with more severe coronary artery obstruction among veterans undergoing cardiac catherization, for blacks but not whites.^38^ A review of discrimination and physical health among black women found few significant associations for indicators of CVD, highlighting the need to better understand the conditions under which the stress of discrimination has adverse health effects.^39^

A 2017 review of 10 longitudinal studies found evidence of a consistent association between self‐reported discrimination and body mass index (BMI), waist circumference, and incidence of obesity.^40^ The associations between experiences of discrimination and adiposity were predominantly linear, and racial discrimination was also significantly associated with changes in BMI and waist circumference among women, but not men. Nonetheless, racial discrimination was significantly associated with the incidence of obesity overall.

Research has also focused on some of the specific pathways that may link exposure to discrimination to changes in health status. A meta‐analysis of discrimination and cortisol output found a small positive association.^41^ Another review of 21 studies of discrimination and the HPA axis found that discrimination has both positive and negative associations with salivary cortisol.^42^ An additional review of 21 studies focused on multisystem responses to discrimination and found strong consistent associations between discrimination and CVD and HPA axis reactivity, but less consistent associations for immune responses.^43^

Another subclinical indicator of heart disease that has been examined in relationship to discrimination is intima‐media thickness (IMT). An early study found that discrimination was positively associated with IMT.^44^ Recent analyses of data from the SWAN study assessed everyday discrimination six times over 10 years and assessed its relationship with intima‐media thickness.^45^ It found that the average levels of discrimination in years 0, 1, 2, 3, 7, and 10 were associated with higher IMT levels at year 12. The association was significant only for white women and not for black, Hispanic, and Chinese women, even though black and Chinese women reported higher levels of discrimination than whites. There is a need to better understand which indicators of discrimination will be predictive of specific health outcomes, for particular population subgroups.

From the earliest studies of discrimination, there has been an increasing interest in the association between discrimination and blood pressure. A recent comprehensive review and meta‐analysis of the association between self‐reported discrimination and hypertension identified 44 studies.^46^ It found a small, significant association between perceived discrimination and hypertension. Larger effect sizes observed were between perceived discrimination and nighttime ambulatory systolic (SBP) and diastolic blood pressure (DBP), especially among blacks. Prior research had found that African Americans are more likely than whites to manifest a blunted blood pressure decline during sleep, a pattern that is predictive of an increased risk for cardiovascular mortality and other outcomes. This review indicated that exposure to discrimination contributes to the decrease in blood pressure dipping during sleep, which results in elevated levels of nighttime blood pressure among blacks. It is currently not clear if the association between discrimination and SBP and DBP is independent of its association with obesity. In the SWAN study, exposure to Everyday Discrimination predicted increases in SBP and DBP over 10 years of follow‐up, even after adjusting for known sociodemographic, behavioral, and medical risk factors. However, consistent across multiple racial groups, when a measure of adiposity (either waist circumference or BMI) was added to the model, the association was no longer significant.^47^

Several recent studies have examined the association between discrimination and inflammation. Among African Americans in the MIDUS study, experiences of discrimination were associated with increased emotional dysregulation (venting and denial) and with increased biological dysregulation, as measured by increases in three indicators of inflammation (interleukin‐6, e‐selectin, and c‐reactive protein).^48^ Another recent study found that lifetime discrimination but not chronic everyday discrimination was associated with increased risk of four markers of inflammation in multivariate models.^49^ Another recent article on discrimination and inflammation found that the associations varied by gender and the indicator of inflammation.^50^

These findings highlight the need to better understand how the different types of discrimination combine to affect health.

Recent analyses have also examined discrimination in relationship to other indicators of biological functioning. Allostatic load (AL) is a measure of multisystem dysregulation. In the MIDUS study, this index sums 24 indicators of risk scores across seven physiological systems.^51^ Analyses of data from African Americans in the MIDUS study found that after adjusting for demographic factors, SES, medication use, cigarette smoking, alcohol use, and mental health symptoms, Everyday Discrimination was associated with higher AL scores. Also, attributions of Everyday Discrimination to race were not more strongly linked to AL than attributions linked to other social statuses. Another recent study has shed light on the pathways that might link discrimination to AL.^52^ In this study, African Americans had higher levels of allostatic load (11 indicators of physiological functioning) and discrimination than their white peers. Discrimination was associated with elevated AL scores. However, this association was fully mediated by measures of anger and poor sleep. Another recent study using national data from the HRS linked higher levels of Everyday Discrimination with lower telomere length for blacks but not whites.^53^

### 4.3 Discrimination and health behaviors

Recent reviews indicate that there is a behavioral pathway linking experiences of discrimination to health, with exposure to discrimination predictive of engaging in more high‐risk behaviors and fewer health‐promoting activities. For example, a 2016 systematic review found 97 studies published between 1980 and 2015 that examined the association between discrimination and alcohol use.^54^ Most studies focused on African Americans and most found positive associations between increased experiences of discrimination, alcohol consumption, and other drinking‐related problems. The review noted that there was considerable variation in quality across the studies and the need for more longitudinal data collection and the use of representative samples. Similarly, a 2019 meta‐analytic review of 27 studies of African Americans found a positive association between discrimination and alcohol consumption, binge drinking, at‐risk drinking, and negative consequences.^55^ Discrimination was unrelated to alcohol use disorder. Earlier reviews found that experiences of discrimination were associated with increased risk of cigarette smoking and drug use.^19^, ^56^

A 2016 review found 17 studies that examined the association between discrimination and sleep (sleep duration and quality), and every study found at least one positive association between exposure to discrimination and poor sleep.^57^ Most studies were cross‐sectional in design (12 of 17); however, three were prospective studies, one was a natural experiment, and one utilized a nine‐day diary component.

### 4.4 Discrimination and health care

Another pathway linking discrimination to poor health status is the potential of experiences of discrimination to lead to reduced health care‐seeking behaviors and adherence to medical regimens. A recent review and meta‐analysis of studies of racism and health service utilization identified 83 papers for review and 59 papers for meta‐analysis.^58^ Major findings included that persons reporting experiences of racial discrimination had two to three times the odds of being less trusting of health care workers and systems, perceiving lower quality of and satisfaction with care, and expressing less satisfaction with patient‐provider communication and relationships. Experiencing racism was also associated with delays in seeking health care and reduced adherence to medical recommendations, although these outcomes were not frequently assessed. Findings related to the use of health services were mixed and mostly not statistically significant. The review also noted important methodological limitations in the research. Many of the measures used to assess discrimination were brief (<25 percent of papers used measures with nine or more items) and over 50 percent of the measures used did not specify a timeframe regarding exposure to racism. A review of 16 qualitative studies examined the role of discrimination in adherence to treatment among persons with HIV.^59^ It was found that exposure to discrimination was associated with less adherence to antiretroviral medication, less self‐care, and lower levels of satisfaction with care.

### 4.5 Discrimination in children and adolescents

Although much of the early research on discrimination and health focused on adult populations, there has been an increasing attention in recent years to the role of discrimination in health outcomes for children and adolescents. A 2013 review identified 121 studies (with 461 outcomes) that examined the association between discrimination and health among persons 0‐18 years old.^60^ Indicators of mental health status were the most frequently assessed. Exposure to discrimination was positively associated with symptoms of anxiety and depression, aggression, internalizing behavior, externalizing behavior, and conduct problems. Discrimination was also inversely associated with indicators of positive mental health, such as life satisfaction, resilience, self‐esteem, and quality of life. Consistent with the literature on adults, a positive association was found between discrimination and poor health practices (alcohol use, drug use, and smoking) in 51 percent of 74 tests. Discrimination was also positively related to poor pregnancy or birth‐related outcomes, such as low birth weight and preterm birth. Research also indicates that adolescents experience discrimination in online contexts. One study, for example, found that after adjustment for age, gender, ethnicity, other adolescent stress, and offline discrimination, online discrimination was positively related to depressive symptoms and anxiety symptoms among 14‐ to 18‐year olds.^61^

A 2018 meta‐analysis of 214 studies examined racial/ethnic discrimination and adolescent outcomes.^62^ It found that there were moderate positive associations between discrimination and multiple indicators of socioemotional distress (eg, depressive symptoms or effects) and internalizing symptoms (eg, anxiety, loneliness, and somatic symptoms). Discrimination was also inversely related to indicators of positive well‐being (eg, life satisfaction, prosocial behaviors, and self‐control), as well as general self‐esteem and self‐worth. The review also included 73 studies that examined the association between discrimination and academic performance. Small‐to‐moderate inverse associations were evident between discrimination and school engagement (eg, attendance), motivation (eg, academic efficacy), and achievement (eg, GPA). This review also documented behavioral pathways among adolescents. There were 71 studies assessing the association between discrimination and risky health behaviors. Small‐to‐moderate positive associations were evident for discrimination with substance abuse, externalizing behaviors (eg, delinquency and anger), affiliation with deviant peers, and risky sexual behaviors (eg, unprotected sex). The analysis also found that for socioemotional distress, associations were stronger for Asian and Latino adolescents compared to African Americans. Another significant moderating effect observed was for the developmental period. Associations with socioemotional distress were stronger in early adolescence (age 10‐13) than late adolescence, and for academics, they were stronger in mid‐adolescence than early adolescence.

A recent study of Latino adolescents illustrates the complex pathways between discrimination and mental health. Using three waves of data, it found that racial/ethnic discrimination predicted increases in symptoms of depression and anxiety.^63^ It also found that outward anger expression was a significant mediator, with greater racial/ethnic discrimination associated with more frequent outward anger expression. Anger expression, in turn, was associated with higher levels of anxiety and depression. This study suggests the possibility that prevention and intervention efforts around managing anger could reduce at least some of the negative effects of racial discrimination on Latino youths' mental health.

A few studies have also reported that adverse effects of discrimination experienced as an adolescent are predictive of physical health outcomes in early adulthood. For example, a study of 331 black adolescents from nine rural counties in Georgia found that youth with high and stable perceived racial discrimination at age 16, 17, and 18 had higher levels of multisystem biological dysregulation as measured by stress hormones (cortisol, epinephrine, and norepinephrine), systolic and diastolic blood pressure, inflammation, and weight by age 20.^64^ A recent review of 30 longitudinal studies found that vicarious discrimination (ie, experiences of discrimination that occur in the life of adults in a child's social network or others with whom the child identify) can adversely affect the health of the target child both prenatally and postbirth.^65^

### 4.6 Discrimination and disparities in health

Most studies of discrimination and health have not examined the contribution that these exposures make to account for racial disparities in health. However, a few studies in the United States and internationally have documented that perceived discrimination makes an incremental contribution over SES in accounting for racial/ethnic inequities in mental health and self‐reported measures of physical health. This pattern has been evident in community and national studies in the United States, New Zealand, Australia, and South Africa.^56^

Recent studies provide further evidence of the role of discrimination in contributing to racial inequities. One study examined SES trajectories over a 33‐year period and their relationship to discrimination and self‐rated health.^66^ It found that increased SES for whites is associated with lower reported discrimination. In contrast, for blacks and Hispanics, upward mobility is associated with increased exposure to discrimination compared to their socioeconomically stable peers. Importantly, exposure to discrimination explained a large part of the black/white gap in self‐rated health (but not the Hispanic/white gap). A study in the United Kingdom also assessed the role of discrimination in ethnic inequalities in mental health.^35^ In cross‐sectional and longitudinal analyses, they found that adjusting for socioeconomic disadvantage and racial discrimination eliminated ethnic inequalities in mental health for some ethnic groups in the United Kingdom but not for others.

### 4.7 Individual and collective protective and resilient responses

Figure 1 also indicates that targets of discrimination are not passive actors but can respond in individual and collective ways to minimize the negative effects of racism. Lewis and colleagues^18^ have reviewed the limited evidence pointing to a number of resources that have been shown to cushion at least some of the negative effects of exposure to discrimination on health. For example, prospective analyses in national studies have shown that religious beliefs and behavior can reduce some of the negative effects of discrimination on health. Other evidence reviewed revealed that there is limited evidence that mindfulness (ie, nonjudgmental attention and awareness) can also reduce the negative effects of discrimination on mental health problems, as measured by depressive symptoms. Finally, research also finds emotional support from family, friends, and supportive professionals can also buffer the adverse impacts of exposure to discrimination on health.

There is still much to be learned about the full range of protective factors that can ameliorate the negative effects of discrimination on health and the conditions that maximize the health‐protective effects of such resources. Relatedly, we need a serious and sustained program of research that would guide us in identifying the interventions that enhance civility and respect for stigmatized groups in our society. There is also a serious need for societal interventions to be developed and implemented to reduce and ultimately eliminate societal prejudice and discrimination. Such research is currently in its infancy.^67^ We also need more systematic attention to the extent to which efforts that seek to comprehensively address the social determinants of health can reduce exposure to racism and its negative consequences.^68^

## 5 DISCUSSION

This review of research on discrimination and health points to many areas that would benefit from further investigation. Prior reviews indicate that methodological limitations that need to be addressed include the overreliance on cross‐sectional studies and refining the measurement approaches to maximize comprehensiveness and accuracy in the assessment of discrimination.^56^ This would require greater attention to capturing the critical stressful dimensions of discriminatory experiences, including the severity, chronicity, and duration of these experiences. There is a need to expand assessment to capture discrimination in multiple domains (eg, race, sex, gender, sexual orientation, stigmatized religious status, and SES), and to extend analyses to assess how exposure in more than one domain relate to each other and combine to affect the adverse impact of discrimination on physical and mental health.^5^ Emerging evidence suggests that utilizing an intersectionality framework that examines associations between discrimination and health, with the simultaneous consideration of multiple social categories, leads to larger associations than when only a single social category is considered.^69^ Given the increasing evidence of the adverse impacts of discrimination early in life, there is also growing awareness of the need to better understand how discriminatory experiences emerge and accumulate over the life course and combine with other stressful experiences to affect physical and mental health.^70^

## 6 CONCLUSION

This article has provided a glimpse of the growing empirical evidence linking self‐reported experiences of discrimination to health. This area of study is only about three decades old. While there is much that we need to learn and important limitations that need to be addressed, the range of health outcomes associated with discrimination is impressive, and the incidence of multiple populations being affected by discrimination, both domestically and globally, is striking. It is now clear that discrimination is a newly emerging risk factor for a broad range of health outcomes that may make an important contribution to understanding racial and ethnic variations in health and health care utilization. This body of research is a reminder that a broad range of psychosocial factors in homes, neighborhoods, workplaces, and schools can be critical determinants of health, and that improving health and reducing inequities in health will likely require interventions outside of the traditional domains of health policy.
